# The effects of *Anethum graveolens* (dill) powder supplementation on clinical and metabolic status in patients with type 2 diabetes

**DOI:** 10.1186/s13063-020-04401-3

**Published:** 2020-06-05

**Authors:** Fatemeh Haidari, Mehrnoosh Zakerkish, Fatemeh Borazjani, Kambiz Ahmadi Angali, Golnaz Amoochi Foroushani

**Affiliations:** 1 Department of Nutrition, Nutrition and Metabolic Diseases Research Center, Ahvaz Jundishapur University of Medical Sciences, Ahvaz, Iran; 2 Health Research Institute, Diabetes Research Center, Ahvaz Jundishapur University of Medical Sciences, Ahvaz, Iran; 3 Department of Biostatistic, School of Health, Ahvaz Jundishapur University of Medical Sciences, Ahvaz, Iran

**Keywords:** Type 2 diabetes, Dill powder, Glycemic control, Lipid profile, Stress oxidative status

## Abstract

**Background:**

The objective of this study was to investigate the effects of *Anethum graveolens* (dill) powder supplementation on glycemic control, lipid profile, some antioxidants and inflammatory markers, and gastrointestinal symptoms in patients with type 2 diabetes.

**Materials and methods:**

In this study, 42 patients with type 2 diabetes were randomly allocated to intervention and control groups and received either 3 g/day dill powder or placebo (3 capsules/day, 1 g each). Fasting blood sugar, insulin, homeostatic model assessment of insulin resistance, lipid profile, high-sensitivity C-reactive protein, total antioxidant capacity, malondialdehyde and gastrointestinal symptoms were measured in all of the subjects at baseline and postintervention.

**Results:**

The dill powder supplementation significantly decreased the mean serum levels of insulin, homeostatic model assessment of insulin resistance, low-density lipoprotein cholesterol, total cholesterol and malondialdehyde in the intervention group in comparison with the baseline measurements (*P* < 0.05). Furthermore, the mean serum levels of high-density lipoprotein and total antioxidant capacity were significantly increased in the intervention group in comparison with the baseline measurement (*P* < 0.05). Colonic motility disorder was the only gastrointestinal symptom whose frequency was significantly reduced by supplementation (*P* = 0.01). The mean changes in insulin, low-density lipoprotein cholesterol, total cholesterol and malondialdehyde were significantly lower in the intervention group than in the control group (*P* < 0.05). In addition, the mean changes in high-density lipoprotein were significantly higher in the intervention group than in the control group (*P* < 0.05).

**Conclusion:**

Dill powder supplementation can be effective in controlling the glycemic, lipid, stress oxidative and gastrointestinal symptoms in patients with type 2 diabetes.

**Trial registration:**

Iran Clinical Trials Registry: IRCT20120704010181N12. Registered on 12 May 2018.

## Introduction

Diabetes is a public health problem that affected 285 million adults in 2010. That number is expected to rise to 439 million—or 7.7% of all adults—by 2030 [1]. In Iran, it has been estimated that 8% of the adult population has diabetes [2]. Major characteristics of type 2 diabetes mellitus (T2DM) are obesity, impaired insulin action, insulin secretory dysfunction and increased endogenous glucose output [3]. Increased free fatty acid flux secondary to insulin resistance is associated with diabetic dyslipidemia, including high plasma triglyceride concentration and low high-density lipoprotein cholesterol (HDL-C) concentration [4]. Inflammatory cytokines contribute to T2DM occurrence by affecting β cell function, which, in turn, promotes the long-term complications of diabetes by intensifying hyperglycemia [5]. Increased glucose uptake by endothelial cells under hyperglycemic conditions also leads to the increased production of free radicals, which decreases antioxidant levels [6]. It is commonly reported that patients with T2DM also encounter gastrointestinal complications, including gastroesophageal reflux disease, gastroparesis, enteropathy, nonalcoholic fatty liver disease and glycogenic hepatopathy [7].

*Anethum graveolens* L (commonly referred to as dill) is a herb commonly used both as a remedy and as a spice [8]. It grows in the Mediterranean region, in Europe, in central and southern Asia, and in the southeastern region of Iran [9]. *A. graveolens* leaves are a source of minerals, proteins and fibers [10]. *A. graveolens* oils are also a source of antioxidants and have antimicrobial and antispasmodic properties [11]. In traditional herbal medicine, *A. graveolens* is used to treat gastrointestinal ailments such as indigestion and flatulence [12]. *A. graveolens* has been shown to have anticancer, antimicrobial, antigastric irritation, anti-inflammatory and antioxidant properties [13]. In diabetic models, the administration of different extractions of *A. graveolens* seed had antioxidant, hypolipidemic and hypoglycemic effects [14].

Earlier studies have reported inconsistent findings regarding the protective effects of *A. graveolens* on the lipid profile and insulin resistance in patients with metabolic syndrome [15, 16]. Randomized clinical trials showed that *A. graveolens* reduced total cholesterol (TC) and low-density lipoprotein cholesterol (LDL-C) but did not change triglyceride or HDL-C levels in patients with T2DM [17]. It has also been reported that *A. graveolens* could have beneficial effects on some inflammatory biomarkers [18] and controversial effects on glucose and insulin [18, 19]. Given the inconclusive results related to glycemic, lipid and inflammatory profiles, it is not clear whether *A. graveolens* helps to increase antioxidants or improve gastrointestinal symptoms. Therefore, the present study was designed to examine the effects of *A. graveolens* powder on the serum levels of glycemic parameters, lipid profile, some antioxidants, inflammatory markers and gastrointestinal symptoms in patients with T2DM.

## Materials and methods

### Study design and participants

**Fig. 1:**
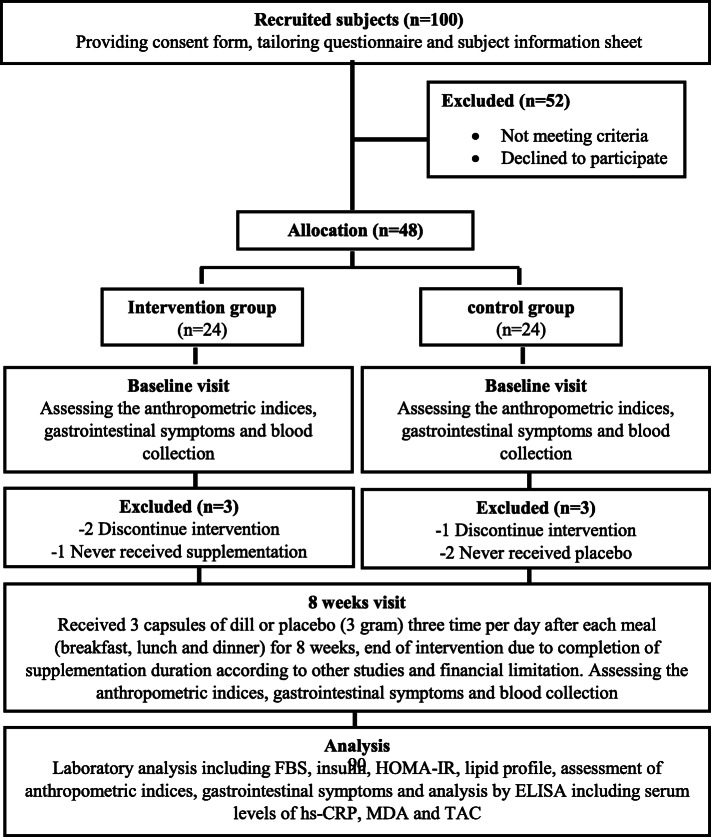
Stages of clinical trial progress. ELISA enzyme-linked immunosorbent assay, FBS fasting blood sugar, HOMA-IR homeostatic model assessment of insulin resistance, hs-CRP high-sensitivity C-reactive protein, MDA malondialdehyde, TAC total antioxidant capacity

A single-center, randomized, double-blind, placebo-controlled study was conducted with 100 patients with T2DM. The patients were recruited from the endocrinology and metabolism clinics of Golestan Hospital at Ahvaz Jundishapur University of Medical Science in Iran between 2017 and 2018 (Fig. 1).

Inclusion criteria were as follows: the patient has diabetes mellitus; is aged 30–60 years; has gastrointestinal symptoms; has a body mass index (BMI) between 25 and 35 kg/m^2^; does not have systemic diseases, thyroid disease, or a kidney disorder; is not pregnant or lactating; and is not taking any dietary supplements or antioxidants, immunosuppressants, or anti-inflammatory agents. Exclusion criteria were the patient shows noticeable changes in the dose of medications and treatment of diabetes, refuses to continue participating in the study, or has less than 90% compliance with the dill capsules.

The diagnosis of T2DM was made according to American Diabetes Association guidelines. Patients with fasting blood sugar (FBS) ≥126 mg/dl, 2-h plasma glucose ≥200 mg/dl, or glycated hemoglobin ≥6.5% were diagnosed with diabetes mellitus [20].

Fifty-two patients did not qualify for this study due to not meeting the inclusion criteria, such as gastrointestinal symptoms and not agreeing to participate. Forty-eight patients were randomly assigned to two groups of intervention (*n* = 24) or placebo (*n* = 24) for 8 weeks. Randomization was performed using computer-generated random numbers by a third person to reduce bias. The third person generated a random block in blocks of 4. The naming of dill or placebo bottles was performed according to random numbers. Odd or even numbers were allocated randomly to groups A or B. A multipart questionnaire including demographic data (age and sex), anthropometric indices, dietary intake, medication, diabetes duration (in years), physical activity and gastrointestinal symptoms was obtained from the subjects. During each visit, every patient was given dill supplement or placebo for 4 weeks, and throughout these weeks the consumption of supplements or placebo by the patients was ensured through telephone calls or text messages. The compliance of patients was checked by counting the remaining capsules. Patients were excluded from the study if they had consumed less than 90% of the prescribed capsules. All participants were asked not to consume dill in their diet during the study. The protocol of this study was approved by the Ethics Committee of Ahvaz Jundishapur University of Medical Sciences (ethical code IR.AJUMS.REC.1396.623) and this study was registered in the Iranian Registry of Clinical Trials website (IRCT20120704010181N12) which is available at http://irct.ir/user/trial/20288/view. Written informed consent was obtained from all participants.

### Supplement and placebo prescription

After confirmation of the *A. graveolens* (dill) herb by the botanist, dried leaves were milled to powder. Capsules containing 1 g dill powder were provided by the Faculty of Pharmacy, Ahvaz Jundishapur University of Medical Sciences. In this study, starch was used as the placebo. The intervention and control groups received either three capsules of 1 g dill or placebo three times per day after each meal (breakfast, lunch and dinner) for 8 weeks. The placebo and dill capsules were matched in terms of size, taste, color and shape.

### Assessment of demographic data, anthropometric indices and food intake

Dietary intake was investigated with a 24-h food recall for 3 days (2 weekdays and 1 weekend day) and dietary intake was analyzed by Nutritionist 4 software specified for Iranian foods. Anthropometric indices (weight, height and BMI) were measured by a trained researcher (nutritionist) at baseline and after the 8-week intervention. Weight (Seca, Germany) was measured while the patients wore light clothing and no shoes at 0.1-kg accuracy. Height was measured using a stadiometer (Seca) with 0.5-cm accuracy without shoes. BMI was calculated (weight in kilograms divided by the square of the height in meters). Physical activity level was evaluated by the Persian form of the International Physical Activity Questionnaire and presented in metabolic equivalent of task (MET) minutes/week. The participants were asked not to change their ordinary dietary intake and physical activity during the intervention.

### Assessment of gastrointestinal symptoms

The assessment of gastrointestinal symptoms was performed by questionnaire at the baseline and at the end of the study [21]. This questionnaire included gastrointestinal symptoms such as gastroesophageal reflux, esophageal motility disorders, dyspepsia, gastric motility disorders and colonic motility disorders.

The numbers 0, 1 and 2 indicate the severity of gastrointestinal symptoms, where 0 = the patient did not have gastrointestinal symptoms, 1 = the patient had occasional gastrointestinal symptoms, and 2 = the patient had permanent gastrointestinal problems.

### Biochemical assays

Fasting blood samples (5 ml) were collected from all participants at the beginning and end of the study and were immediately centrifuged (3000×g, 10 min, 4°C). Blood samples were poured into anticoagulant tubes to extract serum samples and were sent to the laboratory in cool boxes. All samples were stored at −70°C until biochemical analyses. Serum glucose, triglyceride (TG), HDL and TC was measured by standard enzymatic methods using the Pars Azmoun kit (Tehran, Iran). Serum insulin was measured by a human insulin enzyme-linked immunosorbent assay kit (Monobind Inc.). Insulin resistance was estimated according to the homeostatic model assessment of insulin resistance (HOMA-IR), calculated as: HOMA-IR = fasting concentrations of glucose (mg/dl) × fasting insulin (μU/ml) / 405 [22]. The Friedewald formula was used for the calculation of LDL-C [23]:

LDL-C (mg/dl) = TC (mg/dl) − HDL-C (mg/dl) − TG (mg/dl)/5, where TG/5 is used to represent very-low-density lipoprotein cholesterol.

Serum markers of oxidative stress such as total antioxidant capacity (TAC) and malondialdehyde (MDA) were measured by reliable spectrophotometric methods using Zell Bio GmbH kit (Germany). Serum levels of high-sensitivity C-reactive protein (hs-CRP) were measured by enzyme-linked immunosorbent assay kits (Monobind Inc.).

### Outcomes

In this study, LDL-C was considered as the primary outcome. The secondary measurement outcomes were glycemic parameters, other factors of the lipid profile, some antioxidant and inflammatory markers, and gastrointestinal symptoms.

### Statistical analysis

The sample size (95% confidence interval and 80% power) was computed according to the study of Mobasseri et al. [17] and considering LDL-C as the main outcome. Sample size was 21 subjects for each group; 24 subjects were computed in each group allowing for 10% withdrawal. All statistical analyses was performed using SPSS 25. All data were reported as means with standard deviations for quantitative variables or number and percentage for qualitative variables. The normality of the distribution of the data was checked using the Kolmogorov–Smirnov test. A paired sample *t* test was used to compare the results within groups postintervention. An independent sample *t* test was performed to compare the results between the two groups (placebo and intervention). Furthermore, an independent *t* test was used to identify differences between the two groups at the end of the study. The mean changes in variables were calculated using the mean differences of data before and after the study. Analysis of covariance was used to identify any differences between the two groups at the end of the study, adjusting for baseline values and covariates. Furthermore, a chi-squared test was used for statistical analysis of qualitative variables. *P* values less than 0.05 were considered statistically significant in all analyses.

## Results

### Baseline characteristics of the subjects, anthropometric parameters and dietary intake

Overall, 42 patients with T2DM (intervention group, *n* = 21; control group, *n* = 21) completed the study over 8 weeks. The mean age of patients in the intervention and control groups was 50.66 ± 8.22 and 50.42 ± 8.61 years, respectively. No significant differences (*P* ≥ 0.05) were observed in demographic and anthropometric characteristics, duration of diabetes, physical activity or medications between the two groups at baseline (Table 1). No significant differences were observed between the two groups for dietary intake including energy, macronutrients or micronutrients such as antioxidant vitamins C and E at baseline and after the intervention (*P* ≥ 0.05) (Table 2).

**Table 1:** Demographic and anthropometric characteristics of participants at baseline and at the end of the study

Variable	Dill powder (*n* = 21)	Placebo (*n* = 21)	*P* value^a^
Age, years	50.66 ± 8.22	50.42 ± 8.61	0.927
Gender, *n* (%)^b^			
Female	14 (66.7%)	16 (76.2%)	0.495
Male	7 (33.3%)	5 (23.8%)	
Duration of disease, years	8.1 ± 5.49	8.57 ± 6.72	0.714
Weight, kg			
Baseline	78.22 ± 11.08	77.29 ± 8.42	0.761
End	78.08 ± 11.07	77.30 ± 8.25	0.796
*P* value^c^	0.602	0.987	
BMI, kg/m^2^			
Baseline	29.42 ± 3.24	28.95 ± 1.94	0.753
End	29.37 ± 3.29	28.95 ± 1.90	0.725
*P* value^c^	0.627	0.904	
Physical activity, MET minutes/week			
Baseline	1314.33 ± 1036.19	1428.66 ± 1053.76	0.725
End	1254.33 ± 960.52	1495.66 ± 953.10	0.419
*P* value^c^	0.626	0.451	

Values are expressed as means ± standard deviation unless otherwise indicated

*P < 0.05* was considered as significant

*MET* metabolic equivalent of task

^a^Using Mann–Whitney *U* test (for duration of disease and body mass index (BMI)) and independent *t* test (for other variables) between the two groups at baseline and after the intervention

^b^Using chi-squared test

^c^Using Wilcoxon signed rank test (for BMI) and paired *t* test (for other variables)

**Table 2:** Energy, macronutrient and micronutrient intake at baseline and postintervention

Variables	Dill powder (*n* = 21)	Placebo (*n* = 21)	*P* value^a^	*P* value^b^
Energy (kcal)				
Baseline	1881 ± 161	1796 ± 167	0.100	0.113
End	1861 ± 176	1811 ± 163	0.339	0.257
*P* value^c^	0.412	0.593		
Carbohydrate (g)				
Baseline	249.61 ± 23.19	240.40 ± 20.46	0.180	0.146
End	246.51 ± 23.61	238.23 ± 14.28	0.179	0.113
*P* value^c^	0.270	0.536		
Protein (g)				
Baseline	75.41 ± 6.15	73.43 ± 6.09	0.303	0.240
End	74.61 ± 5.72	72.04 ± 6.65	0.187	0.145
*P* value^c^	0.210	0.135		
Fat (g)				
Baseline	61.53 ± 5.37	59.66 ± 4.74	0.241	0.196
End	60.26 ± 6.24	61.93 ± 4.32	0.169	0.098
*P* value^c^	0.147	0.113		
Vitamin A (U)				
Baseline	377.38 ± 103.76	321.32 ± 85.71	0.064	0.034*
End	368.66 ± 115.02	352.93 ± 88.03	0.622	0.693
*P* value^c^	0.779	0.159		
Vitamin C (mg)				
Baseline	87.03 ± 27.87	91.36 ± 29.58	0.628	0.744
End	91.44 ± 30.92	96.85 ± 32.20	0.582	0.633
*P* value^c^	0.592	0.541		
Vitamin E (mg)				
Baseline	2.03 ± 0.72	2.38 ± 0.85	0.166	0.205
End	1.85 ± 0.52	2.12 ± 0.63	0.146	0.136
*P* value^c^	0.171	0.095		

Values are expressed as means ± standard deviation

*P < 0.05 considered as significant, and significant values are shown in bold type

^a^Between-group comparison of variables at baseline and after intervention from independent *t* test (for all variables)

^b^Between-group comparison of variables at baseline and after intervention from analysis of covariance in the adjusted models (adjusted for age, duration of disease and body mass index)

^c^Within-group comparison of variables from paired sample *t* test (for all variables)

### Glycemic control

The results of this study showed that no significant differences were observed in FBS, insulin and HOMA-IR between the two groups at baseline (*P* ≥ 0.05). It was demonstrated that 8 weeks consumption of dill powder significantly decreased the mean serum levels of insulin and HOMA-IR in the intervention group by the end of the study in comparison with the baseline (insulin: 13.27 ± 3.8 vs 10.54 ± 4.51 μU/ml, respectively; *P* = 0.004; HOMA-IR: 4.88 ± 2.37 vs 3.86 ± 2.32, respectively; *P* = 0.039). Furthermore, the insulin levels decreased to a greater extent in the intervention group between the start and the end of the study than in the control group (−2.7 ± 3.83 vs 0.50 ± 4.36, respectively; *P* = 0.015). Analysis of covariance showed that, after adjusting for confounding factors (age, duration of disease, changes in BMI, dietary intake of energy, macronutrients, vitamin A, C and E, and physical activity), the mean changes in insulin were not significantly lower in the intervention group in comparison with the control group after the intervention (Table 3).

**Table 3:** Serum levels of glycemic parameters and lipid profile at baseline and postintervention

Variables	Dill powder (*n* = 21)	Placebo (*n* = 21)	*P* value^a^	*P* value^b^	*P* value^c^	*P* value^d^
FBS (mg/dl)						
Baseline	145.76 ± 50.81	148.61 ± 56.56	0.864	0.883		
End	141.14 ± 40.37	154.23 ± 36.72	0.278	0.623		
*P* value^e^	0.668	0.671				
Difference	−4.61 ± 48.56	5.61 ± 59.76			0.17	0.752
Insulin (μU/ml)						
Baseline	13.27 ± 3.8	11.61 ± 4.91	0.230	0.474		
End	10.54 ± 4.51	12.12 ± 4.23	0.250	0.796		
*P* value^e^	0.004*	0.604				
Difference	−2.7 ± 3.83	0.50 ± 4.36			0.015*	0.05
HOMA-IR						
Baseline	4.88 ± 2.37	4.37 ± 3.01	0.544	0.762		
End	3.86 ± 2.32	4.60 ± 2.02	0.276	0.848		
*P* value^e^	0.039*	0.698				
Difference	−1.02 ± 2.12	0.23 ± 2.68			0.101	0.447
TG (mg/dl)						
Baseline	196.52 ± 60.16	194.66 ± 75.58	0.930	0.626		
End	172.38 ± 69.86	190.19 ± 79.93	0.447	0.664		
*P* value^e^	0.055	0.811				
Difference	−24.14 ± 54.29	−4.47 ± 84.69			0.376	0.343
TC (mg/dl)						
Baseline	160.28 ± 38.26	154.42 ± 32.42	0.596	0.492		
End	149.23 ± 26.7	156.8 ± 32.25	0.412	0.881		
*P* value^e^	0.03*	0.654				
Difference	−11.04 ± 21.7	2.38 ± 23.95			0.064	0.033*
LDL-C (mg/dl)						
Baseline	81.00 ± 34.79	71.71 ± 23.55	0.318	0.516		
End	71.23 ± 26.63	74.80 ± 22.80	0.643	0.357		
*P* value^e^	0.029*	0.325				
Difference	−9.76 ± 19.08	3.09 ± 14.07			0.017*	0.04*
HDL-C (mg/dl)						
Baseline	41.85 ± 11.68	43.14 ± 8.05	0.680	0.939		
End	44.80 ± 9.89	41.76 ± 6.33	0.243	0.343		
*P* value^e^	0.007*	0.185				
Difference	2.59 ± 4.51	−1.38 ± 4.60			0.004*	0.04*

Values are expressed as means ± standard deviation

*HDL-C* high-density lipoprotein cholesterol, *HOMA-IR* homeostatic model assessment of insulin resistance, *LDL-C* low-density lipoprotein cholesterol, *TC* total cholesterol, *TG* triglyceride

*P < 0.05 considered as significant, and significant values are shown in bold type

^a^Between-group comparison of variables at baseline and after intervention from independent *t* test (for all variables)

^b^Between-group comparison of variables at baseline and after intervention from analysis of covariance (ANCOVA) in the adjusted models (adjusted for age, duration of disease, dietary intake of energy, macronutrients, antioxidant vitamins such as vitamins A, C, and E, physical activity and body mass index)

^c^Between-group comparisons mean changes of variables from Mann–Whitney *U* test (for fasting blood sugar (FBS)) and independent *t* test (for other variables)

^d^Between group comparisons mean changes of variables from ANCOVA (adjusted for age, duration of disease, changes of body mass index, dietary intake of energy, macronutrients, vitamins A, C, and E and physical activity)

^e^Within-group comparison of variables, resulted from paired *t* test (for all variables)

### Lipid profile

At baseline, there were no significant differences in the mean serum levels of TG, TC, LDL-C and HDL between the two groups (*P* > 0.05). The dill powder supplementation significantly increased the mean serum levels of HDL in the intervention group in comparison with baseline (44.80 ± 9.89 vs 41.85 ± 11.68 mg/dl, respectively; *P* = 0.007). Furthermore, the mean changes in serum levels of HDL were significantly higher in the intervention group compared to the control group (2.59 ± 4.51 vs −1.38 ± 4.60 mg/dl, respectively; *P* = 0.004). Even after adjusting for confounding factors, there was a significant difference in the mean change in HDL-C between the two groups (*P* = 0.04). In the intervention group, the mean serum levels of LDL-C and TC were significantly decreased postintervention (LDL-C: 81.00 ± 34.79 to 71.23 ± 26.63 mg/dl, respectively; *P* = 0.029; TC: 160.28 ± 38.26 to 149.23 ± 26.7 mg/dl, respectively; *P* = 0.03). Furthermore, the serum levels of LDL-C decreased to a greater extent in the intervention group between the start and the end of the study than in the control group (−9.76 ± 19.08 vs 3.09 ± 14.07 mg/dl, respectively; *P* = 0.017). After adjusting for confounding factors, there was a significant difference in mean change in LDL-C and TC between two groups (*P* = 0.04 and *P* = 0.033, respectively). However, no significant changes were observed in the mean serum levels of TG after the intervention (*P* ≥ 0.05) (Table 3).

### Antioxidant and inflammatory markers

According to the analysis, there were no significant differences in the mean serum levels of hs-CRP, MDA and TAC between the intervention and control groups at baseline (*P* ≥ 0.05). The results of the present study showed that in the intervention group the mean MDA was reduced significantly postintervention in comparison with baseline (3.34 ± 2.05 to 2.22 ± 1.57 μM, respectively; *P* = 0.034). At the end of study, there was a significant difference in the mean changes in MDA between the intervention and control groups both without and with adjusting for confounding factors (−1.11 ± 2.24 vs 0.33 ± 1.62 μM, respectively; *P* = 0.021 vs *P* = 0.013, respectively). Within-group comparison in the intervention group showed that the mean serum levels of TAC significantly increased after 8 weeks of supplementation (0.19 ± 0.05 to 0.25 ± 0.09 mM, respectively; *p* = 0.025). In addition, after the supplementation, the mean serum levels of TAC were significantly higher in the intervention group in comparison with the control group (0.25 ± 0.09 vs 0.16 ± 0.06 mg/dl, respectively; *P* = 0.001). This result for TAC was also observed after adjusting for confounding factors (*P* = 0.004). No significant difference was observed for hs-CRP within and between the two groups (*P* ≥ 0.05) (Table 4).

**Table 4:** The effects of dill supplementation on serum levels of antioxidant and inflammatory markers at baseline and postintervention

Variables	Dill powder (*n* = 21)	Placebo (*n* = 21)	*P* value^a^	*P* value^b^	*P* value^c^	*P* value^d^
MDA (μM)						
Baseline	3.34 ± 2.05	3.72 ± 2.09	0.554	0.886		
End	2.22 ± 1.57	4.06 ± 2.32	0.005*	0.000*		
*P* value^e^	0.034*	0.354				
Difference	−1.11 ± 2.24	0.33 ± 1.62			0.021*	0.013*
TAC (mM)						
Baseline	0.19 ± 0.05	0.17 ± 0.03	0.103	0.137		
End	0.25 ± 0.09	0.16 ± 0.06	0.001*	0.004*		
*P* value^e^	0.025*	0.793				
Difference	0.058 ± 0.11	−0.004 ± 0.7			0.339	0.145
hs-CRP (mg/l)						
Baseline	4.13 ± 0.84	4.29 ± 0.70	0.506	0.388		
End	3.87 ± 0.89	4.32 ± 0.93	0.122	0.143		
*P* value^e^	0.283	0.872				
Difference	−0.25 ± 1.06	0.2 ± 0.8			0.332	0.649

Values are expressed as means ± standard deviation

*hs-CRP* high-sensitivity C-reactive protein, *MDA* malondialdehyde

*P < 0.05 considered as significant, and significant values are shown in bold type

^a^Between-group comparison of variables at baseline and after intervention from independent sample *t* test (for all variables)

^b^Between-group comparison of variables at baseline and after intervention from analysis of covariance (ANCOVA) in the adjusted models (adjusted for age, duration of disease, dietary intake of energy, macronutrients, antioxidant, vitamins such as vitamins A, C and E, physical activity and body mass index)

^c^Between-group comparisons mean changes of variables from Mann–Whitney *U* test (for total antioxidant capacity (TAC)) and independent *t* test (for other variables)

^d^Between-group comparisons mean changes of variables from ANCOVA (adjusted for age, duration of disease, changes of body mass index, dietary intake of energy, macronutrients, vitamin A, C and E and physical activity)

^e^Within-group comparison of variables from paired *t* test (for all variables)

### Gastrointestinal symptoms

Based on the results presented in Table 5, supplementation with dill failed to reduce the frequency of gastrointestinal symptoms such as gastroesophageal reflux, esophageal motility, dyspepsia and gastric motility disorders in comparison with the baseline measurements (*P* ≥ 0.05). Amongst all the symptoms, only colonic motility disorders had their frequency significantly reduced by supplementation (*P* = 0.01), and this decrease was more notable in patients with severe gastrointestinal problems. In the control group, meanwhile, there was no significant reduction in the frequency of gastrointestinal symptoms (*P* ≥ 0.05).

**Table 5:** The effects of dill powder supplementation on gastrointestinal symptoms at baseline and postintervention

	Dill powder (*n* = 21)			Placebo (*n* = 21)		
Gastrointestinal symptom	0	1	2	0	1	2
Gastroesophageal reflux						
Baseline	5 (23.8%)	7 (33.3%)	9 (42.9%)	7 (33.3%)	5 (23.8%)	9 (42.9%)
End	6 (28.6%)	9 (42.9%)	6 (28.6%)	7 (33.3%)	5 (23.8%)	9 (42.9%)
*P* value		0.135			1.00	
Esophageal motility disorders						
Baseline	18 (85.7%)	1 (4.8%)	2 (9.5%)	16 (76.2%)	3 (14.3%)	2 (9.5%)
End	16 (76.2%)	3 (14.3%)	2 (9.5%)	16 (76.2%)	3 (14.3%)	2 (9.5%)
*P* value		0.317			1.00	
Dyspepsia						
Baseline	15 (71.4%)	2 (9.5%)	4 (19%)	14 (66.7%)	3 (14.3%)	4 (19%)
End	12 (57.1%)	7 (33.3%)	2 (9.5%)	13 (61.9%)	5 (23.8%)	3 (14.3%)
*P* value		0.198			0.368	
Gastric motility disorders						
Baseline	12 (57.1%)	1 (4.8%)	8 (38.1%)	11 (52.4%)	2 (9.5%)	8 (38.1%)
End	15 (71.4%)	3 (14.3%)	3 (14.3%)	11 (52.4%)	2 (9.5%)	8 (38.1%)
*P* value		0.112			1.00	
Colonic motility disorders						
Baseline	7 (33.3%)	3 (14.3%)	11 (52.4%)	6 (28.6%)	5 (23.8%)	10 (47.6%)
End	10 (47.6%)	10 (47.6%)	1 (4.8%)	7 (33.3%)	4 (19%)	10 (47.6%)
*P* value		0.010*			0.317	

The numbers 0, 1 and 2 indicate the severity of gastrointestinal symptoms, where 0 = the patient had no gastrointestinal symptoms, 1 = patient had occasional gastrointestinal symptoms, and 2 = the patient had permanent gastrointestinal problems

Data are expressed as percent of relative frequency of gastrointestinal symptoms

*P < 0.05 considered as significant, and significant values are shown in bold type

*P* value within group comparison of variables resulted from chi-squared tests

### Safety, adverse effects and monitoring data

A Data Monitoring Committee supervised this study to detect any possible side effects and to report to the ethics committee of Ahvaz Jundishapur University of Medical Sciences. However, no significant side effects from dill administration were reported in this study.

## Discussion

This study revealed that 8 weeks of supplementation with 3 g/day *A. graveolens* reduced serum insulin and HOMA-IR. Moreover, *A. graveolens* might significantly reduce the serum levels of LDL and TC and enhance HDL when compared to a placebo condition. Patients in the intervention group had low MDA and TAC; however, no significant changes were observed for the serum levels of hs-CRP. In terms of gastrointestinal symptoms, only colonic motility disorders decreased.

These findings are in line with those of several interventional studies confirming the benefits of *A. graveolens* in improving T2DM and metabolic syndrome [14, 16]. The significant reduction in HOMA-IR and serum levels of insulin indicates that *A. graveolens* has a role to play in reducing insulin resistance. Similar beneficial effects of *A. graveolens* on glycemic control have been reported previously. Supplementation of patients with T2DM with 3.3 g/day of *A. graveolens* powder for 8 weeks could significantly reduce levels of insulin [17]. After 6 weeks of supplementation with 1.5 g/day of dill powder tablets, serum levels of FBS were significantly reduced in patients with T2DM [19].

Although Payahoo et al. [18] found a significant decrease in serum levels of insulin, no significant effect was observed for HOMA-IR, which could be due to the reduced levels of FBS in diabetic patients. High antioxidant content (i.e., vitamin C, polyphenols and carotenoids) in *A. graveolens* neutralizes reactive oxygen species and thus plays a role in repairing β cell function and insulin secretion [24, 25].

In this study, serum concentrations of LDL-C and TC decreased, while HDL-C increased significantly at the end of the study. No significant change was seen for serum levels of TG. In agreement with our study, Rashidlamir et al. [26] showed that aerobic training with the use of 2.7 g/day *A. graveolens* resulted in increased HDL and a decreased LDL to HDL ratio in diabetic women compared with the control group; findings for TC, meanwhile, were not statistically significant. In contrast, supplementation with 650 mg *A. graveolens* tablets twice daily increased the serum levels of TG in patients with hyperlipidemia, but no significant changes were seen in TC or LDL [15].

The treatment of hyperlipidemic patients with 1 g/day *A. graveolens* powder for 4 weeks resulted in a significant reduction in the levels of TC, TG, LDL and very-low-density lipoprotein when compared to patients treated with 20 mg/day lovastatin tablets. However, no significant change was observed in the serum levels of HDL [27].

The exact mechanism of the lipid-lowering effects of *A. graveolens* is not yet determined. However, it may relate to the decreased absorption of cholesterol by binding to bile acids, the inhibition of cholesterol and fatty acid synthesis through the suppression of acetyl-CoA carboxylase and HMG-COA reductase activity, and the stimulation of cholesterol clearance by increasing LDL receptors [28–30].

In this study, patients who received 3 g/day *A. graveolens* had lower levels of MDA and higher levels of TAC than patients in the control group, both in crude and adjusted models. MDA is a product of lipid peroxidation and is recognized as an atherogenic agent. Patients with elevated levels of MDA are more susceptible to atherosclerosis, diabetes and other metabolic disorders [31].

Findings from animal studies showed that the administration of different fractions of *A. graveolens* to animals on a high-fat diet decreased their MDA levels and increased the activities of antioxidant enzymes, including superoxide dismutase and catalase. It also increases the levels of glutathione, thus playing a key role in scavenging reactive oxygen species [32]. Hamsters treated with *A. graveolens* extracts or tablets exhibited a significant increase in TAC levels when compared to those on a high-cholesterol diet [33]. *A. graveolens* is composed of a variety of antioxidants, such as flavonoids that are capable of scavenging free radicals [34]. The enhanced levels of antioxidant activity in response to *A. graveolens* might be due to the content of polyphenols and flavonoids. It is possible that normal levels of antioxidants protect individuals against several chronic diseases [35].

We observed a nonsignificant decrease in serum levels of hs-CRP after supplementation with *A. graveolens*. The fact that an increase in bodyweight is an indicator of inflammation [36] could be why a nonsignificant reduction in serum levels of hs-CRP was observed in our study. The anti-inflammatory effects of different forms of *A. graveolens* have been shown in several animal studies [37–39]. Payahoo et al. [18] found a significant decrease in the serum levels of inflammatory biomarkers—including hs-CRP, interleukin-6 and tumor necrosis factor—after 8 weeks of supplementation with 3.3 g dill powder.

In terms of gastrointestinal symptoms, we observed a significant decrease only in colonic motility disorders. It is reported that the most prevalent symptoms among patients with diabetes are colonic motility disorders, which increase with age [21]. The prevalence of gastrointestinal symptoms is positively associated with the duration of diabetes [21, 40]. Patients included in the current study had a mean age of 50 years and a mean disease duration of 8 years—both of which are relatively high. This could be a reason for the observed findings in this regard. Earlier animal models show that *A. graveolens* extract is a potent relaxant of contractions in rat ileum and has antisecretory and antiulcer capabilities as it relates to HCl- and ethanol-induced stomach lesions [41, 42].

To the best of our knowledge, this is the first human study investigating the effects of *A. graveolens* on gastrointestinal symptoms. The major strength of this study was its design as a well-controlled, double-blind clinical trial that controlled for several main confounding factors in different models.

However, there are some limitations to our study. First, this is a single-dose trial, thus preventing any dose-effect associations. It remains unclear whether larger or smaller doses could introduce a stronger clinical effect. Second, the narrow range of inclusion criteria led to unrepresentative samples, therefore limiting the generalizability of the study results to all patients with diabetes. Third, only the data of subjects who completed the study were analyzed; the data of those who were excluded were not measured.

## Conclusion

This study highlights the beneficial effects of *A. graveolens* on insulin resistance, LDL and HDL cholesterol, TC, antioxidant levels and some gastrointestinal symptoms in comparison with a placebo during 8 weeks of supplementation. Further studies are needed to determine molecular levels and to clarify the role of *A. graveolens* in the treatment of diabetes complications.

## Data Availability

The results will not be available before publishing.

## Abbreviations

BMI: Body mass index
FBS: Fasting blood sugar
HDL-C: High-density lipoprotein cholesterol
HOMA-IR: Homeostatic model assessment of insulin resistance
hs-CRP: High-sensitivity C-reactive protein
LDL-C: Low-density lipoprotein cholesterol
MDA: Malondialdehyde
T2DM: Type 2 diabetes mellitus
TAC: Total antioxidant capacity
TC: Total cholesterol
TG: Triglyceride
